# Hypoproteinemia predicts disease severity and mortality in COVID-19: a call for action

**DOI:** 10.1186/s13000-021-01092-5

**Published:** 2021-04-13

**Authors:** Amira Mohammed Ali, Hiroshi Kunugi

**Affiliations:** 1 Department of Mental Disorder Research, National Institute of Neuroscience, National Center of Neurology and Psychiatry, Tokyo, Japan; 2 Department of Psychiatric Nursing and Mental Health, Faculty of Nursing, Alexandria University, Alexandria, Egypt; 3 Department of Psychiatry, Teikyo University School of Medicine, Tokyo, Japan

**Keywords:** Hypoproteinemia, *albumin*, Blood urea nitrogen, Disease severity, Mortality, Coronavirus disease 2019, COVID-19, Multiple organ failure, Intensive care unit, Malnutrition

## Abstract

Proteins represent the major building blocks of body tissues, and they regulate signaling involved in most cellular activities. Coronavirus disease 2019 (COVID-19) infection has been associated with high fatality, especially among older adults. The main cause of death is pulmonary tissue damage and multiple organ failure. The disease is associated with a hypercatabolic state that entails excessive protein loss. This review commentary sheds the light on hypoproteinemia in symptomatic/hospitalized COVID-19 with a special emphasis on its pathophysiology, screening, as well as its contribution to disease severity and adverse effects.

## Introduction

Coronavirus disease 2019 (COVID-19) pathology activates severe protein catabolism resulting in extensive wasting and loss of skeletal muscle mass [1]. Skeletal muscle is a major protein store that contributes to the regulation of metabolism of the whole body [2]. Body protein degradation is evident in severe and critical COVID-19 patients; they manifest low serum levels of total protein, albumin, prealbumin, and high levels of blood urea nitrogen (BUN) [3–6]. BUN is a nitrogenous end product of protein metabolism, and it is associated with mortality in numerous diseases [6]. Protein loss impedes immune functioning and exacerbates symptoms in COVID-19 [3–7].

## Hypoproteinemia and disease outcomes in COVID-19

Critical COVID-19 patients exhibit a crude mortality rate of 49% [6]. The available literature shows that hypoalbuminemia in COVID-19 patients is associated with progression to more severe stages of the illness involving increased need for hospitalization, transfer to intensive care unit (ICU), mechanical ventilation, and nutritional support, in addition to increased mortality [3–5, 7]. In a study comparing blood levels of C-reactive protein, lactate dehydrogenase (LDH), D-dimer, albumin, ferritin, and cardiac troponin T between patients not admitted to the ICU vs ICU admitted and between survivors vs non-survivors, only albumin < 18 g/L and LDH > 731 U/L significantly predicted mortality in an adjusted multivariate analysis while LDH < 425 U/L was associated with lower rates of ICU admission [4].

Low albumin and associated mortality correlate with the cytokine storm, old age, male gender, hospital admission, and comorbid diabetes mellitus [3, 5–7]. Several measures used to predict COVID-19 prognosis depend primarily on albumin level along with some of these characteristics [1, 5]. By using a tree-based machine learning model, Zhou et al. [5] identified the most informative characteristics and hidden interactions that can predict ICU admission in COVID-19 patients: low red blood cell count, male gender, older age, low albumin, low sodium, and prolonged activated partial thromboplastin time (APTT). That model demonstrated precision of 0.91 with an area under the curve (AUC) of 0.92 [5]. Serum albumin may facilitate the detection of malnutrition in COVID-19 when combined with weight changes (e.g., Nutritional Risk Index), total lymphocyte count (e.g., Prognostic Nutritional Index), or with both lymphocyte count and total cholesterol (e.g., the controlling nutritional status score) [2, 8].

Cheng et al. [6] developed a nomogram model based on a combination of initial BUN ≥4.6 mmol/L and D-dimer ≥0.845 μg/mL to identify patients at high risk for in-hospital mortality. The AUC for this model was 0.94 (95% CI 0.90–0.97), with a sensitivity of 85% and specificity of 91% [6]. BUN (adjusted hazard risk (AHR) = 1.06, 95% CI 1.03–1.09; *P* < 0.0001) and D-dimer (AHR = 1.11, 95% CI 1.08–1.14; *P* < 0.0001) remained significantly associated with in-hospital mortality in multivariable analysis adjusted for age, sex, co-morbidity, neutrophil count, lymphocyte count, platelet count, albumin, LDH, procalcitonin, and interleukin-6 [6].

## The pathophysiology of hypoproteinemia in COVID-19

Reasons why hypoproteinemia develops in COVID-19 remain unclear. Anorexia/vomiting and diarrhea, in order, are reported in 25.8 and 29.8% of all COVID-19 patients [6]. Inadequate food supply secondary to these symptoms may direct the body toward protein breakdown for energy production [2]. The degradation of body protein is a result of a complex interaction between inflammatory cytokines, reactive oxygen species, and insulin insensitivity [1, 2]. The inflammatory interactions of the virus that causes COVID-19, severe acute respiratory syndrome-coronavirus-2 (SARS-CoV-2), with human cells induce dysregulation of signaling involved in glucose metabolism resulting in decreased sensitivity to insulin and hyperglycemia signifying inability of the body to use glucose as a source of energy [9]. Hyperglycemia and inflammation potentiate glycation stress, which potentiates the formation of toxic metabolites that accelerate inflammation and oxidative stress (i.e., vicious cycle), eventually leading to damage of cellular protein and lipid structures [2]. Skeletal muscle disuse associated with ICU admission as well as mechanical ventilation accelerate the breakdown of skeletal muscle protein leading to severe hypoproteinemia over a short period of time [1].

Several studies show that hypoproteinemia in COVID-19 is associated with excessive elevation of inflammatory cytokines as well as parameters of coagulopathy (e.g., prolonged APTT, D-dimer, prothrombin time, and activated partial thromboplastin) [3, 5–7]—suggesting that liver damage secondary to hypercytokinemia triggers hypoproteinemia, which furthers inflammation and leads to death [3]. Indeed, liver damage in hospitalized COVID-19 patients is high (58% on admission), and it is associated with multiple organ failure, acute renal damage, increased ICU admission, need for mechanical ventilation, and mortality [10].

## Conclusion

In summary, hypoproteinemia in COVID-19 is associated with the cytokine storm, multiple organ failure, disease severity, and disease-related mortality. Biomarkers of hypoproteinemia (e.g., albumin and BUN) are clearly predictive for COVID-19 outcomes. Clinicians should pay more attention to these markers and intervene accordingly at an early stage, which will prevent progression to severe stages of the illness.

## Data Availability

Not applicable.

## Abbreviations

AHR: Adjusted hazard risk
APTT: Activated partial thromboplastin time
AUC: Area under the curve
BUN: Blood urea nitrogen
COVID-19: Coronavirus disease 2019
ICU: Intensive care unit
LDH: Lactate dehydrogenase
SARS-CoV-2: Severe acute respiratory syndrome-coronavirus-2
